# Mycophenolate Mofetil Attenuates DOCA-Salt Hypertension: Effects on Vascular Tone

**DOI:** 10.3389/fphys.2018.00578

**Published:** 2018-05-18

**Authors:** Arthur D. Moes, David Severs, Koen Verdonk, Nils van der Lubbe, Robert Zietse, A. H. J. Danser, Ewout J. Hoorn

**Affiliations:** ^1^Division of Nephrology and Transplantation, Department of Internal Medicine, Erasmus Medical Center, Erasmus University Rotterdam, Rotterdam, Netherlands; ^2^Division of Pharmacology and Vascular Medicine, Department of Internal Medicine, Erasmus Medical Center, Erasmus University Rotterdam, Rotterdam, Netherlands

**Keywords:** angiotensin II, endothelin-1, salt-sensitive hypertension, sodium transporters, wire myographs

## Abstract

Inflammation is increasingly recognized as a driver of hypertension. Both genetic and pharmacological inhibition of B and T cells attenuates most forms of experimental hypertension. Accordingly, the immunosuppressive drug mycophenolate mofetil (MMF) reduces blood pressure in the deoxycorticosterone acetate (DOCA-) salt model. However, the mechanisms by which MMF prevent hypertension in the DOCA-salt model remain unclear. Recent studies indicate that immunosuppression can inhibit sodium transporter activity in the kidney, but its effect on vascular tone is not well characterized. Therefore, the aim of the present study was to analyze the vascular and renal tubular effects of MMF in the DOCA-salt model in rats (4 weeks without uninephrectomy). Co-treatment with MMF attenuated the rise in blood pressure from day 11 onward resulting in a significantly lower telemetric mean arterial pressure after 4 weeks of treatment (108 ± 7 vs. 130 ± 9 mmHg, P < 0.001 by two-way analysis of variance). MMF significantly reduced the number of CD3^+^ cells in kidney cortex and inner medulla, but not in outer medulla. In addition, MMF significantly reduced urinary interferon-γ excretion. Vascular tone was studied *ex vivo* using wire myographs. An angiotensin II type 2 (AT_2_) receptor antagonist blocked the effects of angiotensin II (Ang II) only in the vehicle group. Conversely, L-NAME significantly increased the Ang II response only in the MMF group. An endothelin A receptor blocker prevented vasoconstriction by endothelin-1 in the MMF but not in the vehicle group. MMF did not reduce the abundances of the kidney sodium transporters NHE3, NKCC2, NCC, or ENaC. Together, our *ex vivo* results suggest that DOCA-salt induces AT_2_ receptor-mediated vasoconstriction. MMF prevents this response and increases nitric oxide availability. These data provide insight in the antihypertensive mechanism of MMF and the role of inflammation in dysregulating vascular tone.

## Introduction

Hypertension is one of the major risk factors for cardiovascular and kidney disease, and therefore an important cause of premature morbidity and mortality. The pathogenesis of hypertension is multifactorial and the result of a complex interplay between dietary and genetic factors, hormones, nerves, and blood pressure regulating organs, including the nervous system, kidneys, heart, and vasculature. Of interest, an increasing body of evidence suggests that inflammation is another driver of hypertension (McMaster et al., 2015; Wenzel et al., 2016; Crowley and Rudemiller, 2017; Foss et al., 2017).

For example, Svendsen (1976) reported that nude mice, which also have genetic aplasia of the thymus, became less hypertensive in response to deoxycorticosterone acetate (DOCA) and salt than haired mice. Similarly, subcutaneous administration of rabbit anti-rat thymocyte serum reduced blood pressure in spontaneously hypertensive rats (SHRs) (Bendich et al., 1981). Intravenous injection of splenic cells from DOCA-salt-treated rats transferred arterial hypertension to recipient rats (Olsen, 1980). Angiotensin II (Ang II)-induced hypertension is attenuated in genetic mouse models lacking B and T lymphocytes (Guzik et al., 2007; Crowley et al., 2010).

Pharmacological inhibition of B and T cells with the immunosuppressive drug mycophenolate mofetil (MMF) can also exert anti-hypertensive effects. Although MMF did not modify hypertension during exogenous Ang II infusion, it did prevent the subsequent development of salt-sensitive hypertension (Rodriguez-Iturbe et al., 2001). MMF did normalize blood pressure in SHRs and Dahl salt-sensitive rats (Rodriguez-Iturbe et al., 2002; Mattson et al., 2006). Similarly, MMF attenuated hypertension and albuminuria in uninephrectomized rats treated with DOCA-salt (Boesen et al., 2010). The anti-hypertensive effects of MMF were not only observed in experimental settings, but also in patients with psoriasis or rheumatoid arthritis treated with MMF (Herrera et al., 2006).

It is incompletely understood how genetic or pharmacological inhibition of B and T cells prevents hypertension. Several studies have shown reduced renal infiltration of lymphocytes and macrophages in MMF-treated hypertensive animals (Rodriguez-Iturbe et al., 2002; Franco et al., 2007, 2013). More recently, such infiltrations have been linked to activation of sodium transporters through direct effects or secretion of cytokines (Kamat et al., 2015; Zhang et al., 2016; Liu et al., 2017). However, few studies have analyzed the effects of immunosuppression on vascular function in experimental hypertension.

Here, we hypothesize that B and T cells mediate experimental hypertension not only through effects on renal sodium transport but also on vascular tone. To do so, we induced hypertension with DOCA-salt in rats and co-treated the animals with MMF or vehicle. Using *ex vivo* myograph analysis of vascular tone, we show that MMF suppresses Ang II type 2 (AT_2_) receptor-mediated vasoconstriction and increases nitric oxide (NO) availability.

## Materials and Methods

### Animal Experiments

The animal protocol was approved by the Animal Care Committee of the Erasmus Medical Center (EMC 3101, # 127-13-01). Sixteen male Sprague-Dawley rats (20 weeks old, average weight 350 g) were obtained from Charles River Laboratories (Sulzfeld, Germany). Animals were randomized to a treatment and a vehicle group (*n* = 8/group). All animals were housed in individual cages and maintained on a 12-h light–dark cycle, having *ad libitum* access to water and standard laboratory rat chow (Na^+^ 0.5%, K^+^ 0.8%). Radiotelemetry transmitters were implanted in eight rats (*n* = 4/group) for continuous blood pressure measurement as described before (van Esch et al., 2010). After a 14-day recovery period, 200 mg 60-day continuous-release DOCA pellets (Innovative Research of America, Sarasota, United States) were implanted subcutaneously in all rats. From this day on, regular drinking water was replaced by 0.9% NaCl for all rats. Rats in the treatment group received a daily dose of 30 mg/kg of MMF (Boesen et al., 2010) via gastric gavage and control rats received an equal volume of water (vehicle). Once a week, animals were placed in metabolic cages for 24 h for the collection of urine and the measurement of food and 0.9% NaCl intake. After 28 days, all animals were killed by left ventricular puncture.

### Biochemical Measurements

Urinary sodium, potassium, and total protein were measured by the Department of Clinical Chemistry of the Erasmus Medical Center. Endothelin-1 (ET-1) in plasma and urine was determined by chemiluminescent ELISA (QuantiGlo, R&D Systems, Abingdon, United Kingdom). A Milliplex cytokine/chemokine immunoassay (Merck Millipore, Billerica, MA, United States) was used for the measurement of interferon-γ (IFN-γ), interleukin-6 (IL-6), chemokine (C-C motif) ligand 5 (CCL5), and tumor necrosis factor-α (TNF-α) in urine.

### Tissue Preparation

After blood collection, kidneys were rapidly excised, weighed, and placed on ice.

The left kidneys of four rats of each group were immersion-fixed in paraformaldehyde and further processed for immunohistochemistry. The right kidneys were used for immunoblotting and were placed in an isolation buffer containing 250 mM sucrose, 10 mM triethanolamine, and a protease inhibitor mix (cOmplete Protease Inhibitor Cocktail Tablets, Roche Diagnostics, Almere, Netherlands) before homogenization. Differential centrifugation of the whole kidney homogenate was performed to obtain a plasma membrane-enriched fraction, as described previously (Ecelbarger et al., 1997; van der Lubbe et al., 2011). The pellet (plasma membrane-enriched fraction) was resuspended in 1 mL of isolation buffer. A total of 60 μL of both fractions was used for a protein quantification assay (Pierce, Thermo Scientific, Rockford, IL, United States) and the remaining samples were stored in 6× Laemmli buffer at -80°C until immunoblotting.

### Immunohistochemistry

Transverse kidney sections were dehydrated and paraffin-embedded, 4-μm sections were cut on a rotary microtome. Staining procedures were performed on the Ventana Benchmark Ultra automatic stainer. For antigen retrieval, deparaffinized sections were incubated with Cell Conditioning 1 medium (Ventana Medical Systems, Tucson, AZ, United States) at 100°C for 32 min. For immunolabeling, slides were incubated in the presence or absence of a polyclonal rabbit anti-CD3ε antibody (1:600, Novus Biologicals, Abingdon, United Kingdom) at 36°C for 32 min, followed by an 8-min incubation with a secondary antibody (Optiview HQ Universal Linker) at 36°C. Detection and visualization was done by subsequent 8-min incubations at 36°C with Optiview HRP Multimer and diaminobenzidine (all from Ventana Medical Systems), after which slides were counterstained with hematoxylin. A rat spleen sample was used as positive control. CD3^+^ cells were counted in 20 randomly selected 400 μm × 200 μm fields in cortex, outer and inner medulla by an investigator blinded to treatment allocation (D.S.). Results are expressed as positive cell counts per mm^2^.

### Immunoblotting

Immunoblotting was performed as described previously (Kim et al., 1998). Antibodies against the following proteins were used: sodium hydrogen exchanger type 3 (NHE3, dilution 1:5000), sodium potassium chloride co-transporter 2 (NKCC2, 1:1000), sodium chloride co-transporter (1:500) and its phosphorylated form at threonine-58 (pNCC, 1:500), and the α- and γ-subunits of the epithelial sodium channel (ENaC, both 1:1000). All antibodies were purchased from StressMarq (Victoria, BC, Canada), except for NCC (Merck Millipore, Billerica, MA, United States) and pNCC (a kind gift by Dr. R. A. Fenton; Pedersen et al., 2010). β-Actin (1:50,000, Abcam) was used for normalization of protein levels. Protein was visualized using horseradish peroxidase-conjugated secondary antibodies (1:3000, Bio-Rad, Veenendaal, Netherlands). Signals were detected by chemiluminescence (Pierce, Rockford, IL, United States), quantified using ImageQuant LAS 4000 (GE Healthcare, Diegem, Belgium), and normalized to actin.

### Wire Myograph Studies

Following isolation and after overnight storage, iliac and mesenteric arteries were cut into segments of approximately 2 mm length and mounted in a Mulvany myograph with separated 6-mL organ baths containing carbogen-gassed Krebs–Henseleit buffer at 37°C. First order mesenteric arteries were used for this experiment, and iliac and mesenteric arteries were used because of their excellent and highly reproducible response to Ang II and ET-1, respectively (Batenburg et al., 2013; Roksnoer et al., 2015). The tension was normalized to 0.9 times the estimated diameter at 100 mmHg of effective transmural pressure. The presence of endothelium was verified by observing relaxation to 1 mM acetylcholine (ACh) after preconstriction with 100 nM of the thromboxane A2 analog U46619 (9,11-dideoxy-11a,9a-epoxy-methanoprostaglandin F2a, Sigma-Aldrich, Zwijndrecht, Netherlands). To determine the maximum contractile response, the tissue was exposed to 100 mM KCl. The segments were then allowed to equilibrate in fresh organ bath fluid for 30 min in the absence or presence of 1 μM of irbesartan, 1 μM of PD123319 (Sigma-Aldrich), 100 μM of NG-nitro-L-arginine methyl ester (L-NAME, Sigma-Aldrich), 1 μM of BQ123 (a selective ET_A_ endothelin receptor antagonist), or 1 μM of BQ788 (a selective ET_B_ endothelin receptor antagonist). Thereafter, concentration–response curves (CRCs) were constructed in response to Ang II (iliac arteries) or ET-1 (mesenteric arteries). ACh-induced relaxation was measured in ET-1 CRCs.

### Statistical Analysis

All data are expressed as means and standard error of the mean. Blood pressure data were analyzed using repeated measures analysis of variance (ANOVA). CRCs were analyzed as described before (MaassenVanDenBrink et al., 1999) to determine the maximum effect (*E*_max_) and pEC_50_ (= -^10^logEC_50_) values. Contractile responses to Ang II or ET-1 are expressed as a percentage of the contraction to 100 mM KCl. Relaxation responses to ACh are expressed as a percentage of the contraction to U46619. CRC and relaxation response data, immunohistochemical CD3^+^ cell quantification data, and log-transformed cytokine/chemokine data were analyzed by one-way ANOVA, with Dunnett’s *post hoc* test where appropriate. *P* < 0.05 was considered statistically significant.

## Results

### Biochemical Responses

**FIGURE 1:**
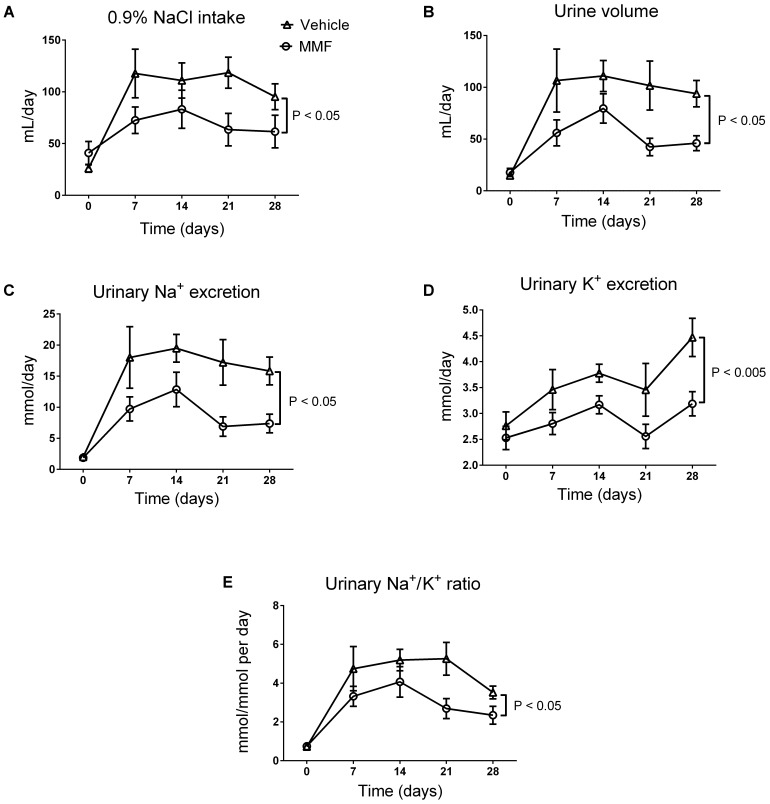
Changes in intake of 0.9% NaCl **(A)**, 24-h urine volume **(B)**, 24-h urinary Na^+^ excretion **(C)**, 24-h urinary K^+^ excretion **(D)**, and the urinary Na^+^/K^+^ ratio **(E)** over the total study period of 28 days of DOCA-salt-treated Sprague-Dawley rats co-treated with MMF or vehicle. Averages ± SEM are shown; *n* = 6–8 animals/group. Measurements were performed once a week.

DOCA-salt induced an increase in fluid intake, diuresis, and urinary Na^+^ and K^+^ excretion, which was sustained throughout the 4-week period (**Figure 1**). Co-treatment with MMF significantly attenuated fluid intake, diuresis, and urinary Na^+^ and K^+^ excretion. Food intake was unaffected by co-treatment with MMF. The body weight at day 28 did not differ between the MMF and vehicle-treated animals, respectively (492 ± 12 vs. 474 ± 16 grams, *P* = 0.4). Median ET-1 levels in serum (0.84 vs. 0.85 pg/mL) and urine (0.09 vs. 0.08 pg/mL) and proteinuria (0.16 vs. 0.14 g/L) were not different between the MMF and vehicle-treated animals, respectively.

### Blood Pressure Response

**FIGURE 2:**
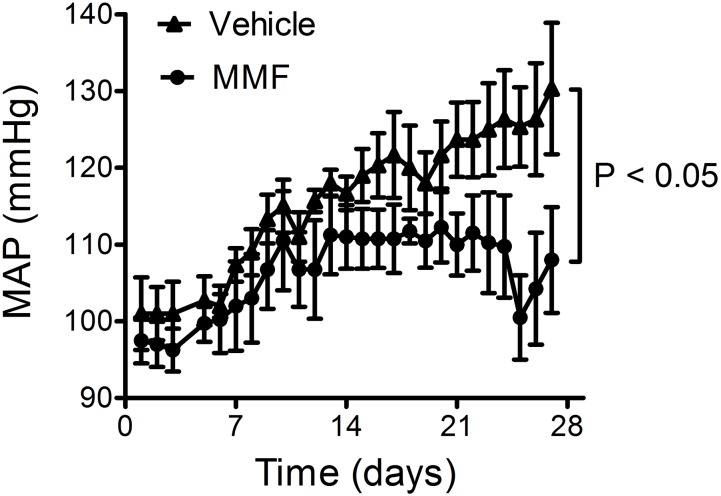
Response of the mean arterial pressure (MAP) to DOCA-salt and co-treatment with MMF or vehicle during the 28-day study period. Averages ± SEM are shown; *n* = 4 animals/group.

DOCA-salt caused a continuing rise in mean arterial pressure (MAP) over the 4-week period (from 101 ± 4 to 130 ± 8 mmHg; **Figure 2**). Co-treatment with MMF attenuated this rise in blood pressure from day 11 onward resulting in a significantly lower MAP after 4 weeks of treatment (108 ± 7 vs. 130 ± 9 mmHg, *P* < 0.001 by repeated measures ANOVA). Co-treatment with MMF did not alter heart rate (303 ± 7 vs. 304 ± 1 beats/min, *P* = 0.8).

### Immunohistochemistry and Urinary Cytokine Excretion

**FIGURE 3:**
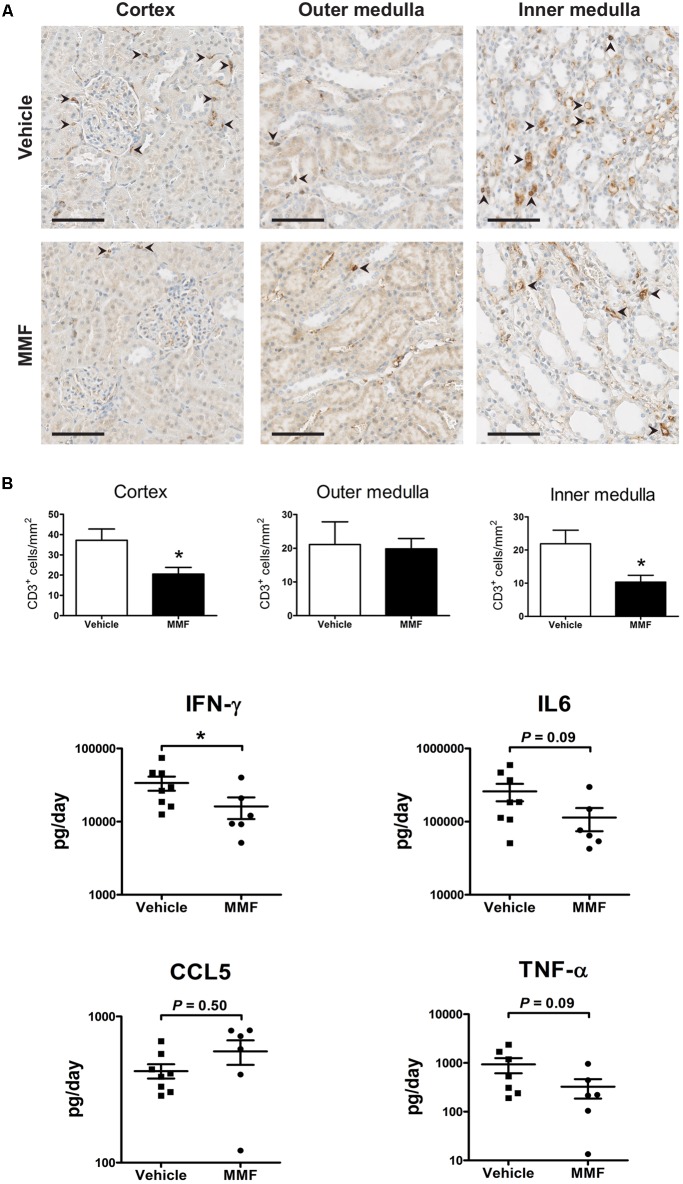
Immunohistochemistry data and urinary chemokines/cytokine excretion. **(A)** Representative images showing localization of CD3C cells (arrowheads) in a kidney section of vehicle- and MMF-treated rats with quantification of CD3C cells in cortex, outer medulla, and inner medulla (scale bar 100 μm), and inner medulla; **(B)** urine cytokine/chemokine excretion. ^∗^*P* < 0.05 vs. vehicle using Student’s *t*-test. IFN-γ, interferon-γ; IL-6, interleukin-6; CCL5, chemokine (C-C motif) ligand 5; TNF-α, tumor necrosis factor-α.

To confirm the anti-inflammatory effect of MMF, we analyzed kidney T-cell infiltration and urinary cytokines (**Figure 3**). MMF significantly reduced the number of CD3^+^ cells in cortex and inner medulla, but not in outer medulla (**Figure 3A**). Urinary IFN-γ excretion was significantly suppressed in MMF-treated animals (**Figure 3B**). No statistically significant differences were found in urinary IL-6, CCL5, and TNF-α between the two groups.

### Vascular Responses

**FIGURE 4:**
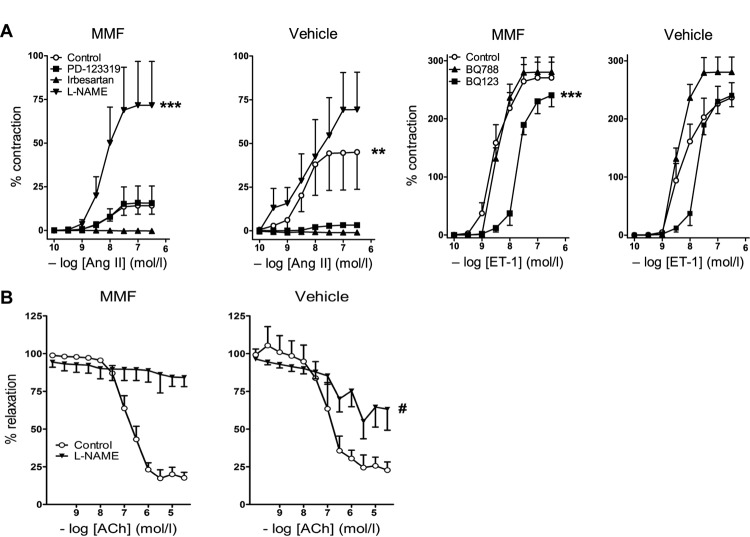
Vascular responses to angiotensin II (Ang II), endothelin-1 (ET-1), and acetylcholine (ACh) in DOCA-salt-treated rats co-treated with MMF or vehicle. **(A)** *Left panel*: responses of iliac arteries to Ang II in the absence (control) or presence of irbesartan, PD123319 or L-NAME. *Right panel*: responses of mesenteric arteries to ET-1 in the absence or presence of BQ123 or BQ788. Contractile responses are expressed as a percentage of the response to 100 mM KCl. **(B)** ACh-induced vasorelaxation. Relaxation responses are expressed as % reduction of preconstriction (with ET-1). Data are mean ± SEM of *n* = 4–8. ^∗∗^*P* < 0.01 and ^∗∗∗^*P* < 0.001 (pEC_50_ or *E*_max_ compared with control), ^#^*P* < 0.001 (*E*_max_ compared with vehicle-treated rats).

Ang II exposure led to a concentration-dependent constriction of iliac arteries of vehicle-treated animals (pEC_50_ = 8.5 ± 0.5, *n* = 7). The *E*_max_ of Ang II in iliac arteries of MMF-treated animals was 15.3 ± 2.4%, compared to 47.4 ± 9.6% of vehicle-treated animals (*P* = 0.007, **Figure 4A**). NO synthase (NOS) inhibition with L-NAME greatly enhanced the Ang II response in the isolated iliac arteries of MMF-treated animals (*E*_max_ = 73.9 ± 12.3, *P* < 0.0001), but the increase was non-significant in control animals (*P* = 0.16). Of note, baseline contractility following preincubation with L-NAME was identical in both groups (data not shown), and thus the differences in contractile response after Ang II are not the consequence of L-NAME alone. The Ang II type 1 (AT_1_) receptor antagonist irbesartan prevented the Ang II-induced constriction in both groups. The AT_2_ receptor antagonist PD123319 blocked the Ang II-induced constriction in iliac arteries of vehicle-treated animals, but not in MMF-treated animals (*P* < 0.001). ET-1 infusion caused a concentration-dependent constriction of mesenteric arteries in vehicle-treated animals (pEC_50_ = 8.3 ± 0.2, *E*_max_ = 235.2 ± 18.7%, *n* = 7). This constriction was unaffected by MMF treatment (**Figure 4A**). ET_A_ receptor inhibition with BQ123 shifted the ET-1 CRC ∼10-fold to the right in mesenteric arteries of MMF-treated animals, but not in vehicle-treated animals (*P* < 0.0001). ACh relaxed mesenteric arteries after preconstriction with ET-1 in vehicle-treated animals (pEC_50_ = 7.0 ± 0.2, *E*_max_ = 22.9 ± 4.4%, *n* = 5). This response was unaltered by MMF treatment (**Figure 4B**). L-NAME completely prevented ACh-induced relaxation in ET-1-preconstricted arteries of MMF-treated animals, but only partially prevented this relaxation in the arteries of vehicle-treated animals (*E*_max_ = 89.0 ± 2.2% vs. 62.7 ± 4.6%, *P* = 0.0009). The latter indicates a diminished NO participation in the ACh response, and thus, given the identical ACh response in both groups, is suggestive for upregulation of non-NO factors to compensate the loss of NO.

### Immunoblotting of Kidney Na^+^ Transporters

**FIGURE 5:**
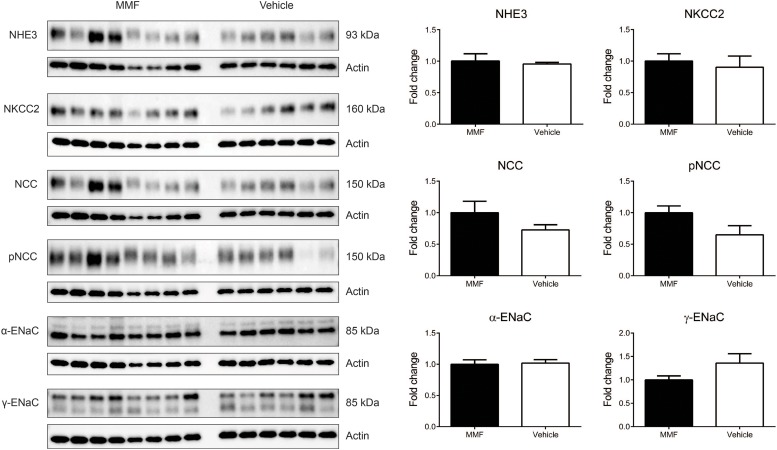
Immunoblots showing protein abundances in of the renal Na^+^ transporters sodium-hydrogen exchanger type 3 (NHE3), Na-K-Cl co-transporter (NKCC2), Na-Cl co-transporter (NCC), NCC phosphorylated at Thr58 (pNCC), α-subunit of the epithelial sodium channel (α-ENaC), and γ-ENaC in whole kidney homogenates of DOCA-salt-treated Sprague-Dawley rats co-treated with MMF or vehicle. No significant differences were identified. Data are mean ± SEM, *n* = 6–8 animals/group.

MMF did not change the abundances of NHE3, NKCC2, NCC, the α- and γ-subunits of ENaC (**Figure 5**). In addition, the abundance of phosphorylated NCC was unchanged.

## Discussion

We show that MMF blunts the development of salt-sensitive hypertension in the DOCA-salt model by reducing vascular resistance, likely through inhibition of T cells. Results of our Mulvany myograph studies point toward a pivotal role for the AT_2_ receptor. Although DOCA-salt is a low Ang II model, a previous study showed that mineralocorticoids regulate vascular Ang II receptors (Schiffrin et al., 1983).

Under normal circumstances, the AT_2_ receptor is known to exert either no effect or a vasodilatory effect via the production of vascular NO (Siragy and Carey, 1997; Batenburg et al., 2004a; Verdonk et al., 2012a). Yet, in isolated arteries of DOCA-salt-treated rats, the AT_2_ receptor appears to have obtained a constrictor function similar to that of the AT_1_ receptor. In isolated arteries of rats receiving DOCA-salt and MMF this AT_2_ receptor-mediated constriction was absent. Moreover, L-NAME fully blocked the ACh-mediated vasodilation after ET-1 preconstriction in the isolated arteries of DOCA-salt + MMF-treated rats, and displayed only a partial blocking effect in the isolated arteries of rats exposed to DOCA-salt alone. Since the ACh response *per se* was the same in both groups, these data suggest that non-NO relaxant mechanisms (e.g., endothelium-dependent hyperpolarizing factor(s); Batenburg et al., 2004b) come into play after DOCA-salt, which are replaced by NO in the presence of MMF. An MMF-induced NO upregulation is also apparent from the huge increase in Ang II response after L-NAME in the MMF treatment group, which did not occur after DOCA-salt alone. Finally, the ET_A_ receptor antagonist BQ123 blocked ET-1-induced constriction in the isolated arteries of MMF-treated rats, and this effect was less apparent in the isolated arteries of rats treated with DOCA-salt only. This is suggestive for ET_A_ receptor upregulation after DOCA-salt. There was no evidence for vasoconstrictor ET_B_ receptor effects after DOCA-salt, since BQ788 did not block ET-1-induced vasoconstriction. Thus, our data do not support constrictor ET_B_ receptor upregulation, although this has been observed earlier in Ang II-dependent hypertensive rat models (Roksnoer et al., 2015).

When studied in young healthy animals, the AT_2_ receptor stimulates vasodilation and natriuresis, thereby counteracting the actions of the AT_1_ receptor, the main mediator of all the well-known deleterious effects of Ang II (Batenburg et al., 2004a; Padia et al., 2006; Verdonk et al., 2012a). However, when studied in older or hypertensive animals, the AT_2_ receptor often loses its vasodilatory actions and instead induces vasoconstriction, similar to the AT_1_ receptor (You et al., 2005; Pinaud et al., 2007; Moltzer et al., 2010). This loss of function might be explained by reactive oxygen species scavenging by NO (Moltzer et al., 2010; Verdonk et al., 2012a). A further possibility is that AT_2_ receptors appear on vascular smooth muscle cells under pathophysiological conditions and act in concert with AT_1_ receptors (e.g., through heterodimerization), as opposed to their predominant, if not exclusive endothelial presence under healthy conditions (van Esch et al., 2006; Moltzer et al., 2010; Verdonk et al., 2012b). We now show that the AT_2_ receptor also loses its vasodilatory effects during DOCA-salt, illustrated by the fact that the Ang II-induced constrictor responses were blocked by PD123319, an AT_2_ receptor antagonist (**Figure 4**). Furthermore, we observed that NOS inhibition with L-NAME greatly enhanced the Ang II contractile response in the MMF-treated animals, whereas the increase after vehicle was non-significant, implying that NOS is preserved in the MMF-treated animals. Indeed, in a rat allograft model, MMF prevented endothelial dysfunction and reduced sensitivity to vasoconstrictors by increasing NO availability through endothelial NOS (Freguin-Bouilland et al., 2011).

Our findings are in line with the results of Guzik et al. (2007), who found a reduced hypertensive response and greater vascular relaxation in Ang II-treated RAG-1^-/-^ mice (which lack B and T cells) than wild-type mice. Furthermore, superoxide production is greater in wild-type than in RAG-1^-/-^ mice after 40 days of DOCA-salt (Guzik et al., 2007). Superoxide is known to react with NO, thereby decreasing NO availability and inducing vasoconstriction (Gryglewski et al., 1986). Guzik et al. (2007) investigated the abundance of the AT_2_ receptor in the aortic wall by Western blot, but found no significant differences between RAG-1^-/-^ and wild type mice. Nevertheless, their previous results and our data support a vasoconstrictive action of the AT_2_ receptor.

Compared with MMF-treated rats, the rats treated with DOCA-salt alone had higher blood pressure, larger volumes of fluid intake and urine output, and greater excretions of Na^+^ and K^+^ (**Figures 1**, **2**). This suggests parallel central effects of DOCA on salt appetite, thirst, and blood pressure, as has been observed previously (Hamlin et al., 1988; Sapouckey et al., 2017). Despite these differences, no changes were observed in any of the renal tubular segments that we studied (**Figure 5**). This is in contrast with recent studies analyzing the role of inflammation on renal sodium transport in hypertensive models. For example, IFN-γ^-/-^ or IL-17A^-/-^ mice had a blunted rise in blood pressure after 2 weeks of high-dose Ang II infusion(Kamat et al., 2015). The IFN-γ^-/-^ mice, but not the IL-17A^-/-^ mice, had a lower abundance of the phosphorylated forms of NKCC2 and NCC than wild-type mice. Furthermore, both IFN-γ^-/-^ and IL-17A^-/-^ mice had lower abundances of NHE3 in the proximal tubules than wild-type mice (Kamat et al., 2015). A study using cortical collecting duct cells of mice found that treatment with IL-6 increased both the mRNA abundance and protein expression of all ENaC subunits (Li et al., 2010). This was accompanied by an increase in the amiloride-sensitive Na^+^ current. These findings suggest that specific parts of the immune system impair pressure natriuresis and enhance distal tubular Na^+^ reabsorption, ultimately leading to hypertension. Our findings also contrast with a recent study by Liu et al. (2017), who found that in DOCA-salt-treated mice, CD8^+^ T cells upregulated NCC expression through direct contact with distal convoluted tubule cells. We propose the following explanations for these differences. First, we used the DOCA-salt model which has low levels of circulating renin and Ang II, in contrast to the Ang II infusion model (Somers et al., 2000). Second, rats received 0.9% NaCl as drinking water, which may have led to a downregulation of kidney Na^+^ transporters. Accordingly, higher NaCl intake in the vehicle-group (with commensurate suppression of the renin–angiotensin system) may have blunted possible differences between groups. Third, we cannot exclude the possibility that changes in renal transporters were only detectable by analyzing the phosphorylated (activated) forms of these proteins. Finally, the duration of our experiment was twice as long as the more commonly used DOCA-salt model, which also includes uninephrectomy. The longer duration of our model may have led to a new steady state with conversion from a volume to a non-volume hypertension mechanism. Thus, in this study, we cannot prove or reject the hypothesis that the anti-hypertensive effect of MMF is also mediated through renal salt handling.

A number of strengths and limitations of this study should be mentioned. The strength of this study is that it is the first to study both vascular resistance and kidney Na^+^ transporters within the same model. A weakness is that we have not yet determined the mechanism of the AT_2_ receptor-mediated constrictor response, the identity of the non-NO component that is upregulated after DOCA-salt, nor the expression of the ET receptors. Finally, future studies should address whether our *ex vivo* observations also hold true *in vivo*. For this, rats should be treated with AT_2_ and ET_A_ receptor antagonists on top of vehicle, DOCA-salt and/or MMF.

## Conclusion

In conclusion, our findings provide additional insight into the pathophysiologic aspects of immune involvement in the development of hypertension. It is known that hypertensive stimuli like old age, high salt diet and atherosclerosis cause stress to the vascular endothelium. This stress causes “sterile inflammation” with activation of both the innate and adaptive immune system, mainly involving infiltration of macrophages and T-cells in the adventitia and perivascular fat with a subsequent production of IL-17, IFN-γ, and TNF-α, amongst others (Epelman et al., 2015; McMaster et al., 2015; Wenzel et al., 2016). These actions induce the production of ROS which results in a decreased bioavailability of NO, impaired vasodilation, and increased vascular stiffness, causing hypertension and ultimately end-organ damage. Our results propose an important role for the AT_2_ receptor, which appears to have a constrictor function under hypertensive stimuli, which is prevented by immunosuppression with MMF.

## Author Contributions

AM, DS, KV, and NvdL conducted the experiments. AM, DS, NvdL, AD, RZ, and EH analyzed the data. AM, DS, AD, and EH wrote the manuscript. All authors reviewed and approved the final version of the manuscript.

## Conflict of Interest Statement

The authors declare that the research was conducted in the absence of any commercial or financial relationships that could be construed as a potential conflict of interest.
